# Prognostic Value of Nutritional Assessments on Overall Survival in Head and Neck Cancer Survivors with Radiation-Induced Brain Necrosis

**DOI:** 10.3390/nu15081973

**Published:** 2023-04-19

**Authors:** Dong Pan, Qingyu Shen, Yi Li, Xiaoming Rong, Honghong Li, Yongteng Xu, Baixuan He, Xuzheng Zuo, Zhenhong Deng, Yamei Tang

**Affiliations:** 1 Department of Neurology, The Eighth Affiliated Hospital, Sun Yat-sen University, Shenzhen 518033, China; dongpan012@hotmail.com; 2 Department of Neurology, Sun Yat-sen Memorial Hospital, Sun Yat-sen University, Guangzhou 510120, China; 3 Guangdong Provincial Key Laboratory of Malignant Tumor Epigenetics and Gene Regulation, Sun Yat-sen Memorial Hospital, Sun Yat-sen University, Guangzhou 510120, China; 4 Guangdong Province Key Laboratory of Brain Function and Disease, Zhongshan School of Medicine, Sun Yat-sen University, 74 Zhongshan 2nd Road, Guangzhou 510080, China

**Keywords:** radiation-induced brain necrosis, head and neck cancer, radiotherapy, survival, malnutrition

## Abstract

Malnutrition is related to worsened prognosis, but the association between nutritional risk status and overall survival in radiation-induced brain necrosis (RN) has never been studied. We included consecutive patients who had received radiotherapy for head and neck cancer (HNC) and subsequently developed RN from 8 January 2005 through to 19 January 2020. The primary outcome was overall survival. We utilized three commonly-used nutritional assessments: the Geriatric Nutritional Risk Index (GNRI), Prognostic Nutritional Index (PNI), and the COntrolling NUTritional Status (CONUT) measure, to quantify the baseline nutritional risk. A total of 398 eligible patients were included. During a median follow-up of 2.3 years, 42 (10.6%) patients died of any cause. Malnutrition at admission was associated with an increased risk of future death, as assessed by the GNRI (per 1-point decreased, HR 1.05, 95%CI 1.02–1.09, *p* = 0.001), the PNI (per 1-point decreased, HR 1.07, 95%CI 1.03–1.12, *p* = 0.002), and the CONUT (per 1-point increased, HR 1.22, 95%CI 1.08–1.37, *p* = 0.001). There were no nonlinear correlations between all three indices and post-RN survival. Among HNC survivors with RN, the assessment of nutritional risk by composite indices upon admission could help identify patients who might be at high risk of future death and deliver better nutritional management.

## 1. Introduction

Radiation-induced brain necrosis (RN) is a severe, irreversible, or even life-threatening cerebral complication after radiotherapy (RT) for cancer patients [1,2]. The incidence of RN was undetermined since the original tumor, treatment, patients’ follow-up periods, and neuroimage criteria were different among various studies, ranging from 5% to 50% [3,4]. However, with the continual improvement in cancer treatment and the development of imaging technology, a higher incidence of RN may be anticipated due to improved survival of cancer patients and earlier detection of brain lesions [5]. Therefore, RN has become a growing healthcare problem among long-term survived cancer patients, and optimal strategies for RN management need to be identified.

Malnutrition is prevalent in cancer patients [6,7], especially in head and neck cancer (HNC) survivors with RN who usually suffer from lower cranial nerves injury and dysphagia [8,9], which may contribute to immune deficiency and worsened prognosis [10,11,12]. However, there are no relevant studies investigating the impact of nutritional risk status when patients are first diagnosed with RN on future death in long-term surviving cancer patients, and little is known about the relationship between nutritional risk status at admission and post-RN survival. In addition, since there are no validated tools for screening the malnutrition status of HNC survivors with RN, it would be meaningful to identify a useful nutritional screening tool for early recognition of patients at high risk of malnutrition to deliver better and more timely nutritional management.

Accordingly, we utilized data from a longitudinal, observational cohort to examine the prognostic value of baseline nutritional risk status assessed by three composite nutritional indices on post-RN survival in long-term surviving HNC patients with newly diagnosed RN. Our secondary goal was to explore which index would be more useful in screening malnutrition among this population.

## 2. Materials and Methods

### 2.1. Study Design and Patients

Since 2005, we have been conducting a longitudinal, observational study on radiotherapy-related nervous system complications at Sun Yat-sen Memorial Hospital, Sun Yat-sen University in Guangzhou, China (NCT03908502). For the current study, we screened all patients admitted to our center from January 2005 through January 2020. Patients were included if they met the following criteria: (i) were aged 18 years or older, (ii) received and completed RT (+/− chemotherapy if applicable) for histologically confirmed HNC, and (iii) had radiographic evidence to support the diagnosis of RN after RT without tumor recurrence or metastases [13]. The diagnosis of RN was based on opinions from both neurologists and radiologists. The diagnostic criteria were as follows: (i) history of RT for HNC, (ii) typical radiographic change of a high-intensity lesion on fluid-attenuated inversion recovery (FLAIR) imaging and a lesion of enhancement on post-gadolinium imaging, especially “soap bubble” or “Swiss cheese” enhancement, irrespective of whether the patients exhibit neurological symptoms or not [14], and (iii) when necessary, the diagnosis was confirmed by positron emission tomography/computed tomography (PET/CT) imaging or biopsy. We excluded patients who: (i) underwent surgical brain necrosis resection before admission to our center, (ii) had undergone RT for brain metastasis, (iii) had unavailable laboratory test data, (iv) had unavailable body height and/or weight data, and (v) had unavailable follow-up data about vital status.

### 2.2. Data Collection

The baseline was defined as the date of RN was first diagnosed. Detailed baseline information was extracted from electronic medical records, including demographic data (date of birth, sex, body height, body weight, cigarette smoking, and alcohol consumption history); prior tumor-related information (TNM stage according to the seventh edition of the AJCC/UICC staging system, tumor progression, the commencement of RT, RT dose and techniques, chemotherapy); medical history (neurological symptoms, co-existing illnesses, and brain surgery); laboratory tests (peripheral lymphocyte count, total cholesterol concentration and serum albumin concentration); and brain MRI assessments. Anti-RN treatment details (corticosteroids, bevacizumab or none) were also collected. Tumor progression was defined by the diagnosis of new metastatic disease or locoregional recurrence after the primary treatment of the patient’s malignancy. Medical records reviewers were not aware of patient outcomes.

### 2.3. Assessments of Baseline Nutritional Risk Status

In this study, we utilized three common-used nutritional indices, the Geriatric Nutritional Risk Index (GNRI), the Prognostic Nutrition Index (PNI), and the COntrolling NUTritional Status (CONUT), all of which can be quickly calculated based on several objective parameters, to quantify the baseline nutritional risk of RN patients, separately.

The Nutritional Risk Index (NRI), first described by Buzby in 1988 [15], combines two nutritional indicators (albumin and weight loss) to quantify the severity of postoperative malnutrition. The GNRI, developed by Bouillanne et al. in 2005 [16], is a modified index derived from the NRI formula replacing the usual weight with the ideal weight, which can be easily calculated by sex and height. The GNRI was originally designed to estimate the risk of morbidity and mortality in hospitalized elderly patients, whose usual weight could not always be provided, by using three objective parameters, serum albumin concentration, body height, and body weight, and the measure has been validated in various diseases. Of note, the GNRI is not an index of malnutrition but rather a “nutritional risk” index that is usually correlated with nutrition-related complications. Since there was no available data on the usual weight of the patients in this retrospective study, we applied the GNRI instead of NRI to score the baseline nutritional status of our subjects. The individual GNRI scores are determined by the following formula:

(1) $$\mathrm{GNRI}=1.489\times \mathrm{serum}\;\mathrm{albumin}\;\left(g/L\right)+41.7\times \frac{\mathrm{body}\;\mathrm{weight}\;\left(\mathrm{kg}\right)}{\mathrm{ideal}\;\mathrm{body}\;\mathrm{weight}\;\left(\mathrm{kg}\right)}$$

Ideal body weight (kg) for men is determined by the following formula:

(2) $$\mathrm{Ideal}\;\mathrm{body}\;\mathrm{weight}\;\left(\mathrm{kg}\right)=\mathrm{height}\;\left(\mathrm{cm}\right)-100-\frac{\mathrm{height}\;\left(\mathrm{cm}\right)-150}{4}$$

Ideal body weight (kg) for women is determined by the following formula:

(3) $$\mathrm{Ideal}\;\mathrm{body}\;\mathrm{weight}\;\left(\mathrm{kg}\right)=\mathrm{height}\;\left(\mathrm{cm}\right)-100-\frac{\mathrm{height}\;\left(\mathrm{cm}\right)-150}{2.5}$$

The actual weight-to-ideal weight ratio in the formula should be one if the actual body weight exceeds the ideal body weight. Lower GNRI scores indicate poorer nutritional status. Patients in the current study were categorized as “Severe risk” (GNRI ≤ 82), “Moderate risk” (82 < GNRI ≤ 92), “Mild risk” (92 < GNRI ≤ 98), and “Absent risk” (GNRI > 98) as suggested in previous studies [17,18].

The PNI, which was originally proposed for relating postoperative outcomes to baseline nutritional status in cancer patients who are undergoing surgery [19,20], can be quickly calculated by two routinely tested parameters, peripheral lymphocyte count and serum albumin concentration. Lower PNI scores indicate higher nutritional risks. As suggested in previous studies, we categorized our patients as “Severe risk” (PNI ≤ 35), “Moderate risk” (35 < PNI ≤ 38), and “Absent risk” (PNI > 38) [21,22]. The individual PNI scores are determined by the following formula:

(4) $$\mathrm{PNI}=5\times \mathrm{peripheral}\;\mathrm{lymphocyte}\;\mathrm{count}\;\left(\times 10^{9}/L\right)+\mathrm{serum}\;\mathrm{albumin}\;\left(g/L\right)$$

The CONUT is an efficient tool for the early detection of in-hospital malnutrition and can be calculated using serum albumin concentration, peripheral lymphocyte count, and total cholesterol concentration [23]. Details of the CONUT scoring system have been described in Table 1. The CONUT scores range from 0 to 12, and a higher score indicates a worse nutritional condition. According to individual CONUT scores, patients were categorized as “Severe risk” (9–12 points), “Moderate risk” (5–8 points), “Mild risk” (2–4 points), and “Absent risk” (0–1 point).

### 2.4. Outcomes

The primary outcome was overall survival, defined as the time from baseline (the first date of the RN diagnosis) to death due to any cause. Patients who were still alive were censored at their last follow-up date. Between July 2020 and March 2021, the vital status (date of death and cause of death, if applicable) of each patient was confirmed through a standardized telephone interview. Telephone interviewers were blinded to individual baseline profiles. All data were censored as of 22 March 2021.

### 2.5. Statistical Analysis

Descriptive analyses and univariable comparisons of baseline characteristics were performed between groups categorized by vital status (alive or dead). Normally contributed continuous variables were described as mean and standard deviation (SD), and the Student *t*-test was used to compare differences between two groups, while those continuous variables that were not conforming to normal distribution were expressed as the median and interquartile range (IQR) and the Mann–Whitney *U* test was used for univariable comparisons. Categorical variables were presented as numbers and percentages and were compared by using *χ*^2^ tests.

The time-to-event analysis for overall survival was conducted using the Cox proportional hazard regression model. We constructed three Cox models to estimate the hazard ratios (HR) of different nutritional risk strata, as well as per the one-point change of the index, on the primary outcome: Model 0 was the crude analysis without confounding adjustment; Model 1 (the main analysis) adjusted for age (continuous), sex (female or male), tumor progression before RN (with or without); lower cranial nerves injury (with or without), bilateral necrosis (with or without), necrosis involving ≥2 brain regions (with or without), and history of stroke (with or without); Model 2 (the sensitivity analysis) additionally adjusted for the time interval from RT to RN diagnosis (continuous), RT techniques (IMRT or non-IMRT), prior TNM stage (stage I, II, III, or IV), having received chemotherapy (with or without), tumor RT dose (continuous) and neck RT dose (continuous) on the basis of –Model 1.

We further performed Restricted Cubic Splines (RCS) analyses adjusted for the same covariates included in Model 1 to examine the potential nonlinear correlations between nutritional risk indices and post-RN survival. Moreover, we compared differences in Kaplan–Meier curves between different nutritional risk groups stratified by optimal cut-off points determined by the maximum *χ*^2^ statistics on post-RN survival in the X-tile software version 3.6.1 (Yale University School of Medicine, New Haven, CT, USA) [24].

All *p* values were reported as two-sided tests with significance defined as *p* < 0.05. Statistical analyses were performed in the R software for macOS (Version 4.0.3, R Core Team, http://www.R-project.org/ [accessed on 19 January 2021]).

## 3. Results

**Figure 1:**
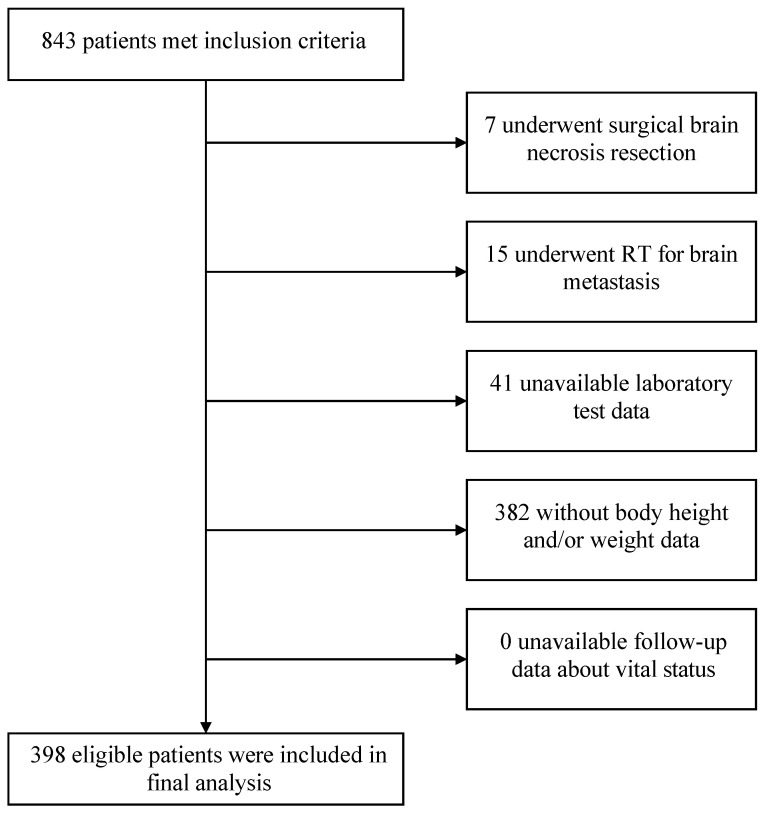
Study flowchart. Abbreviation: RT, radiotherapy.

Of 843 patients consecutively screened, 445 ineligible patients were excluded (7 had undergone surgical brain necrosis resection, 15 underwent RT for brain metastasis, 41 had unavailable laboratory test data, and 382 without body height and/or weight data). In total, 398 patients were finally involved in the analysis (Figure 1).

### 3.1. Cohort Characteristics

Over a median follow-up period of 2.3 years, 42 (10.6%) patients died of any cause. Cohort characteristics were displayed in Table 2, and the actual sample size of each variable with missing data was noted. Dead patients, as compared with alive patients, were older when being first diagnosed with RN (median age [IQR], 53.8 [45.9–60.2] years vs. 50.6 [43.9–56.6] years, *p =* 0.08), more often with lower cranial nerves injury (27/42 [64.3%] vs. 150/356 [42.1%], *p* = 0.01), more severe RN lesions on brain MRI (necrosis involving ≥2 brain regions, 18/42 [42.9%] vs. 55/356 [15.4%], *p* < 0.001), less often receiving IMRT (10/27 [37.0%] vs. 146/234 [62.4%], *p* = 0.02), lower BMI (mean ± SD, 20.5 ± 3.0 kg/m^2^ vs. 21.7 ± 3.2 kg/m^2^, *p* = 0.03), lower peripheral lymphocyte count (median [IQR], 1.0 [0.8–1.2] × 10^9^/L vs. 1.2 [0.9–1.6] × 10^9^/L, *p =* 0.01), lower total cholesterol concentration (median [IQR], 182 [159–205] mg/dL vs. 197 [168–223] mg/dL, *p =* 0.07), and lower serum albumin concentration (median [IQR], 34.4 [28.2–38.3] g/L vs. 36.8 [30.2–40.7] g/L, *p =* 0.09). Dead patients were more likely to have a poorer nutritional condition at admission: lower GNRI score (median [IQR], 86.9 [78.3–95.5] points vs. 93.2 [83.4–99.9] points, *p* = 0.02), lower PNI score (median [IQR], 38.9 [34.0–43.3] points vs. 42.8 [37.1–47.3] points, *p* = 0.02) and higher CONUT score (median [IQR], 4.0 [2.0–6.0] points vs. 3.0 [1.0–5.0] points, *p =* 0.01). The baseline characteristics of 398 patients stratified by three nutritional risk indices are displayed in Tables S1–S3.

### 3.2. Nutritional Risk Status on Post-RN Survival Using Cox Regression Models

We labeled patients as “Severe risk”, “Moderate risk”, “Mild risk” (only in the GNRI and the CONUT assessments), and “Absent risk” according to individual nutritional risk scores as suggested in the previous studies, and constructed Cox regression models to estimate the hazard ratios (HR) of baseline nutritional risk on the primary outcome. The Kaplan–Meier plots of the probability of post-RN survival in different nutritional risk strata assessed by the three indices are reported in Figures S1–S3. Table 3 shows the relationship between the three nutritional indices and all-cause mortality. Compared with “Absent risk”, the baseline “Moderate-to-Severe risk” was associated with worse post-RN survival, both as assessed by the GNRI (Model 1, Moderate risk: HR 4.19, 95%CI 1.44–12.21, *p* = 0.009; Severe risk: HR 4.43, 95%CI 1.58–12.40, *p* = 0.005; Per 1-point decreased: HR 1.05, 95%CI 1.02–1.09, *p* = 0.001), the PNI (Model 1, Moderate risk: HR 2.92, 95%CI 1.17–7.30, *p* = 0.02; Severe risk: HR 2.65, 95%CI 1.19–5.89, *p* = 0.02; Per 1-point decreased: HR 1.07, 95%CI 1.03–1.12, *p* = 0.002), or the CONUT (Model 1, Moderate risk: HR 3.72, 95%CI 1.33–10.37, *p* = 0.01; Severe risk: HR 4.67, 95%CI 1.33–16.37, *p* = 0.02; Per 1-point decreased: HR 1.22, 95%CI 1.08–1.37, *p* = 0.001). In the sensitivity analysis, we additionally included tumor- and RT-related covariates in the Cox model (Model 2). Thus, 139 patients were excluded due to missing data. After confounding adjustment in Model 2, both the GNRI (Model 2, Moderate risk: HR 5.62, 95%CI 1.23–25.80, *p* = 0.03; Severe risk: HR 5.59, 95%CI 1.30–23.96, *p* = 0.02; Per 1-point decreased: HR 1.06, 95%CI 1.01–1.10, *p* = 0.01) and the PNI assessments (Model 2, Per 1-point decreased: HR 1.08, 95%CI 1.01–1.15, *p* = 0.02) showed that baseline malnutrition was still significantly associated with an increased risk of future death, while the CONUT assessment failed to yield similar results.

### 3.3. Examination of Nonlinear Associations and the Optimal Cut-off Points of All Three Nutritional Risk Indices

**Figure 2:**
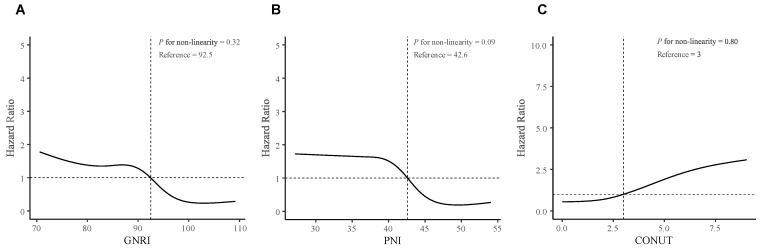
Restricted Cubic Spline analyses for testing non-linearity associations. (**A**) GNRI; (**B**) PNI; (**C**) CONUT. The RCS analyses were adjusted for age (continuous), sex (female or male), tumor progression before RN (with or without); lower cranial nerves injury (with or without), bilateral necrosis (with or without), necrosis involving ≥ 2 brain regions (with or without), and history of stroke (with or without). The solid lines represent the hazard ratios, and the gray zones represent the 95% confidence interval. Abbreviations: RN, Radiation-induced brain necrosis; GNRI, Geriatric Nutritional Risk Index; PNI, Prognostic Nutritional Index; CONUT, Controlling Nutritional Status.

**Figure 3:**
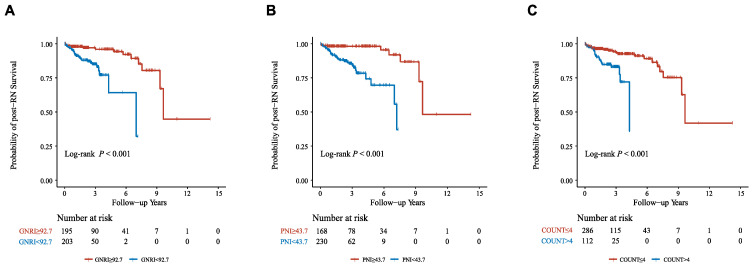
Nutritional risk stratifications and post-RN survival. (**A**) GNRI; (**B**) PNI; (**C**) CONUT. Abbreviations: GNRI, Geriatric Nutritional Risk Index; PNI, Prognostic Nutritional Index; CONUT, Controlling Nutritional Status.

We further performed RCS analyses to determine whether there was a potential nonlinear correlation between baseline nutritional risk scores and post-RN survival. In RCS analyses (Figure 2), we did not find nonlinear associations between the three indices and the primary outcome (Nonlinear tests, for GNRI: *p* = 0.32; for PNI: *p* = 0.09; for CONUT: *p* = 0.80). Moreover, The X-tile software generated optimal cut-off points of the three nutritional risk indices to classify patients into “low risk” (GNRI ≥ 92.7, PNI ≥ 43.7, or CONUT 0–4) or “high risk” (GNRI < 92.7, PNI < 43.7, or CONUT 5–12), and showed favorable discriminations (All log-rank *p* < 0.001; Figure 3, Figures S4–S6).

## 4. Discussion

Although malnutrition had been reported to be related to adverse clinical outcomes in various diseases, such as heart failure and acute ischemic stroke [17,25], whether there would be such an association between nutritional risk status and overall survival among long-term survived HNC patients with RN had never been studied. To the best of our knowledge, this is the first study to examine the prognostic value of baseline nutritional assessments on post-RN survival. In this study, we found that the baseline nutritional risk status assessed by the GNRI, the PNI, and the CONUT was an independent predictive factor for post-RN survival after adjusting for other clinical risk factors, suggesting that malnutrition at the time when patients were first diagnosed with RN was significantly associated with an increased risk of all-cause death in long-term survived HNC patients.

Our previous study had identified five independent risk factors for post-RN survival, including age, tumor progression before RN, lower cranial nerves injury, bilateral necrosis, and history of stroke [26], which had all been adjusted in the Cox model in the current study. Although a well-performed prediction model involving those five predictors has been established to quantify the risk of post-RN death, it remains difficult to reduce post-RN deaths by modulating these risk factors since most of them are unmodifiable factors. Therefore, it is imperative to identify a novel predictor capable of predicting and improving survival by intervening in it. Encouragingly, in the current study, after adjusting for significant risk factors (Model 1), we found that the higher baseline nutritional risk status was still significantly associated with higher mortality, and the sensitivity analysis yielded similar results (Model 2), suggesting that the malnutrition at admission, assessed by three nutritional indices, respectively, was an independent predictor of post-RN survival. Furthermore, nutritional status is a modifiable risk factor, and we can improve the survival outcomes of RN patients through better nutrition management. Hence, our study will have an important implication for clinicians regarding the optimal strategies for long-term survived cancer patients suffering from RN.

Malnutrition is very common in patients who have completed the RT and/or chemotherapy courses for HNC [8]. As for RN patients, in addition to brain necrosis that can lead to cognitive impairment, mood disorders, and subsequent anorexia and emaciation, they always simultaneously suffer from radiation damage to salivary glands, pharyngeal muscles, and lower cranial nerves, resulting in dysphagia and eating disorders [9,27]. Thus, malnutrition is even more prevalent and severe among this population. In this study, we utilized three simple, common-used nutritional indices: the GNRI, the PNI, and the CONUT, to evaluate the baseline nutritional conditions of study participants, all of which were easily calculated based on several routinely assessed parameters in clinical practice (serum albumin concentration, peripheral lymphocyte count, total cholesterol concentration, and body weight and height) and performed satisfactorily to predict the risk of post-RN death. The study results showed that our patients had poorer nutritional conditions, given the median GNRI in our study was only 92.4 and the proportion of patients with moderate-to-severe risk reached 48.5%, which was obviously higher than that in the previous studies on long-term cared elderly (mean GNRI 96.5 and 18.3% with moderate-to-severe risk) [28], and patients with heart failure with preserved ejection fraction (median GNRI 99.8 and 11.2% with moderate-to-severe risk) [17]. The assessments of the PNI and the CONUT also suggested higher nutritional risk in our population as compared with that reported in the previous studies on patients with admission to the coronary critical care unit and those with acute ischemic stroke [21,22,25]. Considering that the median age was only 50.9 years in our studied population, the malnutrition in long-term survived HNC patients suffering from RN could be much more severe than we thought, and clinicians should pay more attention to nutritional assessment and nutritional management of these patients.

Despite these significant results, there is abundant room for further progress in the nutritional assessment among HNC survivors with RN. First, the present study was a retrospective analysis based on an ongoing, prospective, observational cohort, and our results were mainly derived from available data, which were almost routinely collected data in clinical practice in the database. Thus, the choice of nutritional risk status screening tools was limited. Many useful nutritional tools could not be applied in the current study due to limited data, such as the Malnutrition Screening Tool (MST), Creatinine Height Index (CHI), Bioelectrical impedance analysis (BIA), and Nutritional Risk Screening 2002 (NRS-2002). In the future, we believe that these more comprehensive measures of the nutritional risk status by combining multi-domain assessments will be worth carrying out to make a more precise judgment of the individual nutritional risk status of patients with RN. Second, future studies will be more meaningful if the laboratory values of vitamins (C, D, E, K, thiamine, B6, B12, and folic acid) and trace elements (zinc, selenium, and iron) could be included in to determine the nutritional status and their relevance to the prognosis of RN. However, the blood levels of vitamins and trace elements are not routinely tested in the present cohort. Hence, we are unfortunately unable to assess the associations of these markers with malnutritional status as well as clinical outcomes due to insufficient data. In addition, some medications would have an impact on the nutritional status. The first-line treatment for RN includes corticosteroids and bevacizumab. Long-term use of corticosteroids is expected to be harmful to patients’ nutritional status, such as osteoporosis, muscle loss, and fat gain, while there are no such effects being observed in bevacizumab use. On the other hand, corticosteroids may also improve individual nutritional status by improving dysphagia due to lower cranial nerve injuries (patients would be able to eat better), so the effect of corticosteroids on the nutritional status of RN patients cannot be concluded at the moment, and this unsolved problem warrants future study.

Our study showed that the GNRI had superior prognostic value to the other two indices since both the main and the sensitivity analyses yielded robust results, and its calculation only required three simple parameters, namely serum albumin concentration and body weight and height. Therefore, we would recommend clinicians routinely assess each patient’s GNRI, rather than the PNI and the CONUT, in clinical practice for screening the individual nutritional risk status. Subsequent assessment following Global Leadership Initiative on Malnutrition (GLIM) criteria for diagnosis and severity grading of malnutrition [29], when weight loss, BMI, and muscle mass can be measured, would also be recommended, with the etiologic criteria being the disease (HNC survivors with RN), to deliver timely nutritional interventions. However, future prospective studies would still be needed to determine the effect of nutritional intervention based on GNRI-based two-step evaluation following GLIM criteria in HNC survivors with RN.

### Strengths and Limitations of the Study

To the best of our knowledge, this is the first study to examine the prognostic value of nutritional risk status, as assessed by multiple nutritional risk indices at admission, on overall survival after being diagnosed with RN in long-term survived HNC patients. Most studies had been looking for risk factors contributing to the development of RN, but little attention had been paid to post-RN clinical outcomes. This cohort study revealed an independent predictor on post-RN survival among HNC survivors, suggesting that the nutritional risk status of patients at the time of their first RN diagnosis was significantly associated with the risk of future death, which would help to improve the prognosis of RN patients by identifying those who might be at higher risk of death and could benefit from timely nutritional interventions.

There were several limitations in this study as well. First of all, it was an observational, retrospective study based on a single-center cohort in China, and we only included HNC survivors. Thus, there might be selection bias. Second, some patients had unavailable baseline physical measurements and laboratory test data and were subsequently excluded from the analysis, which might have led to some confounding bias. For instance, the database did not report weight and height in 382/843 patients (~45%), and the exclusion of these subjects might result in some potential biases. Third, the GNRI, PNI, and CONUT assessments were not conducted during post-discharge outpatient follow-up. Therefore, we were unable to investigate the association of longitudinal changes in nutritional status with long-term outcomes. Finally, we failed to obtain enough data to analyze the impact of baseline nutritional status on the incidence of future nutrition-related complications, such as bedsores and infections. Further prospective studies would be needed to validate our findings.

## 5. Conclusions

Among HNC survivors with RN, malnutrition was associated with a higher risk of future death. The assessment of nutritional risk by composite indices upon admission could help identify patients who may be at high risk of death and deliver better nutritional management. Further prospective, randomized studies would be needed to determine whether the nutritional intervention could improve overall survival in long-term cancer survivors suffering from RN.

## Figures and Tables

**Table 1:** Details of the CONUT scoring system.

**Serum albumin, g/L**	**level**	≥35	30–34.9	25–29.9	<25
	**point**	0	2	4	6
**Total cholesterol, mg/dL**	**level**	≥180	140–179	100–139	<100
	**point**	0	1	2	3
**Lymphocyte count, ×109/L**	**level**	≥1.60	1.20–1.59	0.80–1.19	<0.80
	**point**	0	1	2	3
**CONUT score**	**point**	0–1	2–4	5–8	9–12
	**Risk strata**	Absent risk	Mild risk	Moderate risk	Severe risk

Abbreviations: CONUT, controlling nutritional status score.

**Table 2:** Cohort characteristics.

Baseline Characteristics	All	Alive	Dead	*p-*Values
	N = 398	N = 356	N = 42	
Sex—male, *n* (%)	291 (73.1%)	256 (71.9%)	35 (83.3%)	0.16
Age—yrs, median (IQR)	50.9 (44.5–57.0)	50.6 (43.9–56.6)	53.8 (45.9–60.2)	0.08
Follow-up period—yrs, median (IQR)	2.3 (1.1–3.6)	2.3 (1.2–3.6)	1.4 (0.7–3.3)	0.02
GNRI—point, median (IQR)	92.4 (83.1–99.5)	93.2 (83.4–99.9)	86.9 (78.3–95.5)	0.02
GNRI strata				0.10
>98—Absent risk	128 (32.2%)	119 (33.4%)	9 (21.4%)	
>92, ≤98—Mild risk	77 (19.3%)	71 (19.9%)	6 (14.3%)	
>82, ≤92—Moderate risk	104 (26.1%)	92 (25.8%)	12 (28.6%)	
≤82—Severe risk	89 (22.4%)	74 (20.8%)	15 (35.7%)	
PNI—point, median (IQR)	42.6 (36.5–47.2)	42.8 (37.1–47.3)	38.9 (34.0–43.3)	0.02
PNI strata				0.03
>38—Absent risk	278 (69.8%)	256 (71.9%)	22 (52.4%)	
>35, ≤38—Moderate risk	38 (9.5%)	31 (8.7%)	7 (16.7%)	
≤35—Severe risk	82 (20.6%)	69 (19.4%)	13 (31.0%)	
CONUT—point, median (IQR)	3.0 (2.0–5.0)	3.0 (1.0–5.0)	4.0 (2.0–6.0)	0.01
CONUT strata				0.05
0~1—Absent risk	99 (24.9%)	92 (25.8%)	7 (16.7%)	
2~4—Mild risk	187 (47.0%)	171 (48.0%)	16 (38.1%)	
5~8—Moderate risk	90 (22.6%)	76 (21.3%)	14 (33.3%)	
9~12—Severe risk	22 (5.5%)	17 (4.8%)	5 (11.9%)	
Height—cm, mean (SD)	165.4 (7.3)	165.2 (7.3)	166.9 (7.0)	0.15
Weight—kg, mean (SD)	59.1 (10.7)	59.3 (10.9)	57.1 (8.4)	0.12
BMI—kg/m^2^, mean (SD)	21.5 (3.2)	21.7 (3.2)	20.5 (3.0)	0.03
Tumor progression before RN—Yes, *n* (%)	47 (11.8%)	40 (11.2%)	7 (16.7%)	0.31
Lower cranial nerves injury—Yes, *n* (%)	177 (44.5%)	150 (42.1%)	27 (64.3%)	0.01
Hypertension—Yes, *n* (%)	47 (11.8%)	41 (11.5%)	6 (14.3%)	0.61
Diabetes—Yes, *n* (%)	15 (3.8%)	14 (3.9%)	1 (2.4%)	>0.99
Stroke—Yes, *n* (%)	33 (8.3%)	29 (8.1%)	4 (9.5%)	0.77
Cigarette Smoking—Yes, *n* (%)	57 (14.3%)	52 (14.6%)	5 (11.9%)	0.81
Alcohol consumption—Yes, *n* (%)	24 (6.0%)	20 (5.6%)	4 (9.5%)	0.30
Laboratory tests—median (IQR)				
Red blood cells—×10^9/L	4.4 (4.1–4.8)	4.4 (4.1–4.8)	4.5 (4.1–4.7)	0.93
Hemoglobin—g/L	129.1 (16.5)	129.6 (16.0)	124.6 (19.5)	0.12
White blood cells—×10^9/L	5.6 (4.5–7.0)	5.6 (4.4–6.9)	6.2 (4.7–7.6)	0.15
Neutrophils—×10^9/L	3.7 (2.7–5.2)	3.7 (2.7–5.0)	4.6 (3.2–5.6)	0.01
Lymphocyte—×10^9/L	1.2 (0.9–1.5)	1.2 (0.9–1.6)	1.0 (0.8–1.2)	0.01
Total cholesterol—mg/dL	196 (167–222)	197 (168–223)	182 (159–205)	0.07
Albumin—g/L	36.7 (30.1–40.5)	36.8 (30.2–40.7)	34.4 (28.2–38.3)	0.09
Brain MRI assessment—*n* (%)				
Bilateral necrosis	181 (45.5%)	158 (44.4%)	23 (54.8%)	0.27
Involving ≥2 brain regions	73 (18.3%)	55 (15.4%)	18 (42.9%)	<0.001
Anti-RN treatment—*n* (%)				
Corticosteroids	176 (44.2%)	157 (44.1%)	19 (45.2%)	>0.99
Bevacizumab	84 (21.1%)	78 (21.9%)	6 (14.3%)	0.34
None of the above	168 (42.2%)	148 (41.6%)	20 (47.6%)	0.56
Time from RT to RN—yrs, median (IQR)	3.4 (2.5–6.2) (*n* = 283)	3.4 (2.5–6.1) (*n* = 254)	3.4 (3.0–8.0) (*n* = 29)	0.21
TNM stage—*n* (%)				0.78
I	5/272 (1.8%)	5/244 (2.0%)	0/28 (0.0%)	
II	22/272 (8.1%)	20/244 (8.2%)	2/28 (7.1%)	
III	138/272 (50.7%)	121/244 (49.6%)	17/28 (60.7%)	
IV	107/272 (39.3%)	98/244 (40.2%)	9/28 (32.1%)	
RT technique—IMRT, *n* (%)	156/261 (59.8%)	146/234 (62.4%)	10/27 (37.0%)	0.02
Tumor RT dose—Gy, mean (SD)	69.1 (7.6) (*n* = 266)	69.2 (6.9) (*n* = 239)	68.7 (12.6) (*n* = 27)	0.84
Neck RT dose—Gy, mean (SD)	54.0 (22.8) (*n* = 278)	54.2 (22.6) (*n* = 250)	52.4 (25.1) (*n* = 28)	0.73
Chemotherapy—*n* (%)	231/277 (83.4%)	209/249 (83.9%)	22/28 (78.6%)	0.43
Neoadjuvant	123/277 (44.4%)	112/249 (45.0%)	11/28 (39.3%)	0.71
Concurrent	190/277 (68.6%)	175/249 (70.3%)	15/28 (53.6%)	0.11
Adjuvant	18/277 (6.5%)	15/249 (6.0%)	3/28 (10.7%)	0.41

Abbreviations: GNRI, Geriatric Nutritional Risk Index; PNI, Prognostic Nutritional Index; CONUT, Controlling Nutritional Status; BMI, Body Mass Index; SD, Standard deviation; IQR, Interquartile range; RT, Radiotherapy; RN, Radiation-induced brain necrosis; IMRT, Intensity-modulated radiation therapy; MRI, Magnetic resonance imaging.

**Table 3:** Univariable and multivariable Cox regression of the impact of nutritional status on all-cause mortality.

NUTRITIONAL INDICES		Model 0		Model 1		Model 2	
		Unadjusted-HR (95% CI)	*p-*Values	Adjusted-HR (95% CI)	*p-*Values	Adjusted-HR (95% CI)	*p-*Values
**GNRI**							
Absent risk	(GNRI > 98)	[Ref]	··	[Ref]	··	[Ref]	··
Mild risk	(92 < GNRI ≤ 98)	1.56 (0.55–4.40)	0.40	1.38 (0.47–4.07)	0.56	1.06 (0.39–6.52)	0.51
Moderate risk	(82 < GNRI ≤ 92)	4.45 (1.67–11.85)	0.003	4.19 (1.44–12.21)	0.009	5.62 (1.23–25.80)	0.03
Severe risk	(GNRI ≤ 82)	6.33(2.37–16.88)	<0.001	4.43 (1.58–12.40)	0.005	5.59 (1.30–23.96)	0.02
GNRI per 1-point decreased		1.06 (1.03–1.09)	<0.001	1.05 (1.02–1.09)	0.001	1.06 (1.01–1.10)	0.01
**PNI**							
Absent risk	(PNI > 38)	[Ref]	··	[Ref]	··	[Ref]	··
Moderate risk	(35 < PNI ≤ 38)	3.80 (1.55–9.30)	0.004	2.92 (1.17–7.30)	0.02	3.80 (0.97–14.97)	0.06
Severe risk	(PNI ≤ 35)	3.56 (1.65–7.66)	0.001	2.65 (1.19–5.89)	0.02	2.16 (0.70–6.64)	0.18
PNI per 1-point decreased		1.09 (1.04–1.14)	<0.001	1.07 (1.03–1.12)	0.002	1.08 (1.01–1.15)	0.02
**CONUT**							
Absent risk	(CONUT 0–1)	[Ref]	··	[Ref]	··	[Ref]	··
Mild risk	(CONUT 2–4)	1.33 (0.55–3.24)	0.53	1.40 (0.56–3.52)	0.47	1.79 (0.57–5.62)	0.32
Moderate risk	(CONUT 5–8)	4.12 (1.55–10.92)	0.004	3.72 (1.33–10.37)	0.01	3.33 (0.85–13.06)	0.09
Severe risk	(CONUT 9–12)	6.11 (1.83–20.36)	0.003	4.67 (1.33–16.37)	0.02	3.25 (0.61–17.43)	0.17
COUNT per 1-point increased		1.26 (1.12–1.40)	<0.001	1.22 (1.08–1.37)	0.001	1.16 (0.99–1.36)	0.06

**Model 0** (the unadjusted analysis) was a crude analysis without confounding adjustment. **Model 1** (the main analysis) was adjusted for age (continuous), sex (female or male), tumor progression before RN (with or without), lower cranial nerves injury (with or without), bilateral necrosis (with or without), necrosis involving ≥2 brain regions (with or without), and history of stroke (with or without). **Model 2** (the sensitivity analysis) was adjusted for Model 1, and the time interval from RT to RN diagnosis (continuous), RT techniques (IMRT or non-IMRT), prior TNM stage (stage I, II, III, or IV), having received chemotherapy (with or without), tumor RT dose (continuous) and neck RT dose (continuous). A total of 259 patients were included in Model 2 (139 patients were excluded due to missing data). Abbreviations: GNRI, Geriatric Nutritional Risk Index; PNI, Prognostic Nutritional Index; CONUT, Controlling Nutritional Status; HR, Hazard Ratio.

## Data Availability

Data supporting the findings of the study are available on reasonable request after approval of a proposal from the corresponding author (Y.T.).

## Supplementary Materials

The following supporting information can be downloaded at: https://www.mdpi.com/article/10.3390/nu15081973/s1, Table S1. Baseline characteristics grouped by GNRI strata; Table S2. Baseline characteristics grouped by PNI strata; Table S3. Baseline characteristics grouped by CONUT strata; Figure S1. Baseline GNRI and post-RN survival; Figure S2. Baseline PNI and post-RN survival; Figure S3. Baseline CONUT and post-RN survival; Figure S4. The optimal cut-off value of GNRI is determined by the X-tile software; Figure S5. The optimal cut-off value of PNI is determined by the X-tile software; Figure S6. The optimal cut-off value of CONUT is determined by the X-tile software; Supplementary analysis. The prognostic value of the baseline BMI in predicting mortality in head and neck cancer survivors with radiation-induced brain necrosis.
