# Supplementary material for: Prognostic Value of Nutritional Assessments on Overall Survival in Head and Neck Cancer Survivors with Radiation-Induced Brain Necrosis
Source: Nutrients. 2023 Apr 19;15(8):1973. doi: 10.3390/nu15081973 (PMC10141744; doi:10.3390/nu15081973)

Prognostic value of nutritional assessments on overall survival in head and neck cancer survivors with radiation-induced brain necrosis

DP[1], QS[1], YL[1], XR, HL, YX, BH, XZ, ZD, YT*

Supplements

Table S1. Baseline characteristics grouped by GNRI strata.

Table S2. Baseline characteristics grouped by PNI strata.

Table S3. Baseline characteristics grouped by CONUT strata.

Figure S1. Baseline GNRI and post-RN survival.

Figure S2. Baseline PNI and post-RN survival.

Figure S3. Baseline CONUT and post-RN survival.

Figure S4. The optimal cut-off value of GNRI determined by the X-tile software.

Figure S5. The optimal cut-off value of PNI determined by the X-tile software.

Figure S6. The optimal cut-off value of CONUT determined by the X-tile software.

Supplementary analysis. The prognostic value of the baseline BMI in predicting mortality in head and neck cancer survivors with radiation-induced brain necrosis.

---

1 These authors contributed equally to the study.

* Correspondence to: YT, tangym@mail.sysu.edu.cn

Table S1. Baseline characteristics grouped by GNRI strata.

	**All Patients**	**Absent risk**	**Mild risk**	**Moderate risk**	**Severe risk**	***P* values**
Sample size – n	**398**	**128**	**77**	**104**	**89**	
Sex – males, n (%)	291 (73.1%)	101 (78.9%)	60 (77.9%)	58 (55.8%)	72 (80.9%)	<0.001
Age – yrs, median (IQR)	50.9 (44.5-57.0)	49.4 (43.9-54.4)	52.2 (46.2-57.5)	50.6 (44.5-58.5)	52.6 (45.4-58.4)	0.167
Follow-up period– yrs, median (IQR)	2.3 (1.1-3.6)	2.7 (1.4-5.8)	1.6 (0.9-4.5)	2.2 (1.0-3.1)	2.5 (1.7-2.9)	<0.001
Presence of primary endpoint – n (%)	42 (10.6%)	9 (7.0%)	6 (7.8%)	12 (11.5%)	15 (16.9%)	0.104
GNRI – point, median (IQR)	92.4 (83.1-99.5)	102.0 (99.8-105.6)	95.1 (93.6-96.3)	86.3 (84.1-89.3)	76.3 (72.4-79.9)	<0.001
PNI – point, median (IQR)	42.6 (36.5-47.2)	47.9 (46.6-50.7)	43.8 (42.5-46.5)	39.2 (36.6-41.7)	31.4 (29.4-34.7)	<0.001
PNI strata						<0.001
>38 – Absent risk	278 (69.8%)	128 (100.0%)	77 (100.0%)	65 (62.5%)	8 (9.0%)	
>35, ≤38 – Moderate risk	38 (9.5%)	0 (0.0%)	0 (0.0%)	24 (23.1%)	14 (15.7%)	
≤35 – Severe risk	82 (20.6%)	0 (0.0%)	0 (0.0%)	15 (14.4%)	67 (75.3%)	
CONUT – point, median (IQR)	3.0 (2.0-5.0)	2.0 (1.0-2.0)	2.0 (1.0-3.0)	4.0 (3.0-5.0)	6.0 (5.0-8.0)	<0.001
CONUT strata						.
0~1 – Absent risk	99 (24.9%)	60 (46.9%)	29 (37.7%)	10 (9.6%)	0 (0.0%)	
2~4 – Mild risk	187 (47.0%)	68 (53.1%)	47 (61.0%)	60 (57.7%)	12 (13.5%)	
5~8 – Moderate risk	90 (22.6%)	0 (0.0%)	1 (1.3%)	34 (32.7%)	55 (61.8%)	
9~12 – Severe risk	22 (5.5%)	0 (0.0%)	0 (0.0%)	0 (0.0%)	22 (24.7%)	
Height – cm, mean (SD)	165.4 (7.3)	166.2 (7.1)	165.4 (7.1)	163.3 (7.0)	166.7 (7.4)	0.004
Weight – kg, mean (SD)	59.1 (10.7)	64.3 (9.7)	58.6 (10.4)	55.2 (9.8)	56.6 (10.6)	<0.001
BMI – kg/m^2, mean (SD)	21.5 (3.2)	23.2 (2.4)	21.4 (3.1)	20.6 (3.0)	20.4 (3.5)	<0.001
Tumor progression before RN – Yes, n (%)	47 (11.8%)	10 (7.8%)	10 (13.0%)	15 (14.4%)	12 (13.5%)	0.394
Lower cranial nerves injury – Yes, n (%)	177 (44.5%)	45 (35.2%)	35 (45.5%)	49 (47.1%)	48 (53.9%)	0.045
Hypertension – Yes, n (%)	47 (11.8%)	17 (13.3%)	11 (14.3%)	11 (10.6%)	8 (9.0%)	0.670
Diabetes – Yes, n (%)	15 (3.8%)	4 (3.1%)	5 (6.5%)	4 (3.8%)	2 (2.2%)	0.544
Stroke – Yes, n (%)	33 (8.3%)	8 (6.2%)	9 (11.7%)	9 (8.7%)	7 (7.9%)	0.591
Cigarette Smoking – Yes, n (%)	57 (14.3%)	20 (15.6%)	11 (14.3%)	15 (14.4%)	11 (12.4%)	0.928
Alcohol consumption – Yes, n (%)	24 (6.0%)	10 (7.8%)	6 (7.8%)	2 (1.9%)	6 (6.7%)	0.173
Laboratory tests – median (IQR)						
Red blood cells – ×10^9/L	4.4 (4.1-4.8)	4.6 (4.3-5.0)	4.5 (4.2-4.9)	4.3 (3.9-4.7)	4.3 (3.9-4.6)	<0.001
Hemoglobin – g/L	129.1 (16.5)	135.6 (12.9)	130.5 (14.7)	125.4 (16.7)	122.8 (18.9)	<0.001
White blood cells – ×10^9/L	5.6 (4.5-7.0)	5.7 (4.5-7.1)	5.6 (4.4-7.5)	5.6 (4.4-6.5)	5.4 (4.5-7.3)	0.799
Neutrophils – ×10^9/L	3.7 (2.7-5.2)	3.8 (2.8-5.7)	3.8 (2.7-5.0)	3.7 (2.8-4.6)	3.6 (2.7-5.4)	0.776
Lymphocyte – ×10^9/L	1.2 (0.9-1.5)	1.2 (0.9-1.7)	1.2 (1.0-1.4)	1.1 (1.0-1.5)	1.2 (0.9-1.5)	0.489
Total cholesterol – mg/dL	196.0 (167.0-222.0)	209.0 (183.8-227.2)	187.0 (168.0-227.0)	193.5 (165.0-215.5)	177.0 (149.0-212.0)	<0.001
Albumin – g/L	36.7 (30.1-40.5)	41.8 (40.1-43.8)	37.7 (36.6-39.7)	33.0 (30.2-35.4)	25.9 (23.4-28.5)	<0.001
Brain MRI assessment – n (%)						
Bilateral necrosis	181 (45.5%)	62 (48.4%)	41 (53.2%)	38 (36.5%)	40 (44.9%)	0.128
Involving ≥2 brain regions	73 (18.3%)	22 (17.2%)	11 (14.3%)	18 (17.3%)	22 (24.7%)	0.327
Anti-RN treatment – n (%)						
Corticosteroids	176 (44.2%)	78 (60.9%)	38 (49.4%)	34 (32.7%)	26 (29.2%)	<0.001
Bevacizumab	84 (21.1%)	41 (32.0%)	14 (18.2%)	14 (13.5%)	15 (16.9%)	0.003
None of the above	168 (42.2%)	28 (21.9%)	33 (42.9%)	58 (55.8%)	49 (55.1%)	<0.001

	All Patients	Absent risk	Mild risk	Moderate risk	Severe risk	*P* values
Sample size – n	**398**	**128**	**77**	**104**	**89**	
Time from RT to RN – yrs, median (IQR)	3.4 (2.5-6.2) (*n*=283)	3.3 (2.5-5.6) (*n*=105)	3.8 (2.2-7.7) (*n*=52)	3.3 (2.6-5.0) (*n*=66)	3.6 (3.0-7.3) (*n*=60)	0.627
TNM stage – n (%)						.
I	5/272 (1.8%)	1/102 (1.0%)	3/50 (6.0%)	0/63 (0.0%)	1/57 (1.8%)	
II	22/272 (8.1%)	9/102 (8.8%)	5/50 (10.0%)	3/63 (4.8%)	5/57 (8.8%)	
III	138/272 (50.7%)	50/102 (49.0%)	23/50 (46.0%)	37/63 (58.7%)	28/57 (49.1%)	
IV	107/272 (39.3%)	42/102 (41.2%)	19/50 (38.0%)	23/63 (36.5%)	23/57 (40.4%)	
RT technique – IMRT, n (%)	156/261 (59.8%)	60/98 (61.2%)	24/50 (48.0%)	43/61 (70.5%)	29/52 (55.8%)	0.101
Tumor RT dose – Gy, mean (SD)	69.1 (7.6) (*n*=266)	70.0 (2.7) (*n*=100)	67.1 (9.6) (*n*=52)	69.9 (4.5) (*n*=60)	68.6 (12.6) (*n*=54)	0.129
Neck RT dose – Gy, mean (SD)	54.0 (22.8) (*n*=278)	58.2 (17.8) (*n*=102)	50.6 (23.6) (*n*=54)	54.8 (23.3) (*n*=63)	48.9 (27.8) (*n*=59)	0.050
Chemotherapy – n (%)	231/277 (83.4%)	88/102 (86.3%)	36/54 (66.7%)	56/63 (88.9%)	51/58 (87.9%)	0.003
Neoadjuvant	123/277 (44.4%)	53/102 (52.0%)	21/54 (38.9%)	27/63 (42.9%)	22/58 (37.9%)	0.254
Concurrent	190/277 (68.6%)	71/102 (69.6%)	29/54 (53.7%)	48/63 (76.2%)	42/58 (72.4%)	0.053
Adjuvant	18/277 (6.5%)	8/102 (7.8%)	2/54 (3.7%)	5/63 (7.9%)	3/58 (5.2%)	0.749

Abbreviations: GNRI, Geriatric Nutritional Risk Index; PNI, Prognostic Nutritional Index; CONUT, Controlling Nutritional Status; BMI, Body Mass Index; SD, Standard deviation; IQR, Interquartile range; RT, Radiotherapy; RN, Radiation-induced brain necrosis; IMRT, Intensity-modulated radiation therapy; MRI, Magnetic resonance imaging.

Table S2. Baseline characteristics grouped by PNI strata.

	All Patients	Absent risk	Moderate risk	Severe risk	*P* values
Sample size – n	**N=398**	**N=278**	**N=38**	**N=82**	
Sex – males, n (%)	291 (73.1%)	202 (72.7%)	25 (65.8%)	64 (78.0%)	0.353
Age – yrs, median (IQR)	50.9 (44.5-57.0)	50.3 (44.0-56.0)	53.4 (44.8-56.7)	53.5 (45.9-60.8)	0.074
Follow-up period– yrs, median (IQR)	2.3 (1.1-3.6)	2.2 (1.1-4.4)	2.4 (1.2-3.0)	2.5 (1.5-2.9)	0.421
Presence of primary endpoint – n (%)	42 (10.6%)	22 (7.9%)	7 (18.4%)	13 (15.9%)	0.025
GNRI – point, median (IQR)	92.4 (83.1-99.5)	97.4 (91.6-101.6)	83.4 (80.2-85.9)	77.0 (72.4-81.0)	<0.001
GNRI strata					<0.001
>98 – Absent risk	128 (32.2%)	128 (46.0%)	0 (0.0%)	0 (0.0%)	
>92, ≤98 – Mild risk	77 (19.3%)	77 (27.7%)	0 (0.0%)	0 (0.0%)	
>82, ≤92 – Moderate risk	104 (26.1%)	65 (23.4%)	24 (63.2%)	15 (18.3%)	
≤82 – Severe risk	89 (22.4%)	8 (2.9%)	14 (36.8%)	67 (81.7%)	
PNI – point, median (IQR)	42.6 (36.5-47.2)	45.1 (42.2-48.1)	36.5 (35.7-37.2)	31.2 (29.3-33.1)	<0.001
CONUT – point, median (IQR)	3.0 (2.0-5.0)	2.0 (1.0-3.0)	4.5 (4.0-5.0)	7.0 (6.0-9.0)	<0.001
CONUT strata					.
0~1 – Absent risk	99 (24.9%)	99 (35.6%)	0 (0.0%)	0 (0.0%)	
2~4 – Mild risk	187 (47.0%)	167 (60.1%)	19 (50.0%)	1 (1.2%)	
5~8 – Moderate risk	90 (22.6%)	12 (4.3%)	19 (50.0%)	59 (72.0%)	
9~12 – Severe risk	22 (5.5%)	0 (0.0%)	0 (0.0%)	22 (26.8%)	
Height – cm, mean (SD)	165.4 (7.3)	165.4 (7.1)	163.9 (8.1)	166.1 (7.2)	0.316
Weight – kg, mean (SD)	59.1 (10.7)	59.3 (10.9)	55.1 (9.8)	60.1 (10.1)	0.048
BMI – kg/m^2, mean (SD)	21.5 (3.2)	21.6 (3.2)	20.5 (3.2)	21.8 (3.1)	0.106
Tumor progression before RN – Yes, n (%)	47 (11.8%)	31 (11.2%)	4 (10.5%)	12 (14.6%)	0.677
Lower cranial nerves injury – Yes, n (%)	177 (44.5%)	118 (42.4%)	23 (60.5%)	36 (43.9%)	0.109
Hypertension – Yes, n (%)	47 (11.8%)	34 (12.2%)	4 (10.5%)	9 (11.0%)	0.968
Diabetes – Yes, n (%)	15 (3.8%)	12 (4.3%)	1 (2.6%)	2 (2.4%)	0.909
Stroke – Yes, n (%)	33 (8.3%)	21 (7.6%)	5 (13.2%)	7 (8.5%)	0.471
Cigarette Smoking – Yes, n (%)	57 (14.3%)	43 (15.5%)	5 (13.2%)	9 (11.0%)	0.581
Alcohol consumption – Yes, n (%)	24 (6.0%)	17 (6.1%)	2 (5.3%)	5 (6.1%)	1.000
Laboratory tests – median (IQR)					
Red blood cells – ×10^9/L	4.4 (4.1-4.8)	4.5 (4.2-4.9)	4.3 (3.7-4.5)	4.3 (3.9-4.7)	<0.001
Hemoglobin – g/L	129.1 (16.5)	131.5 (15.6)	121.8 (15.9)	124.2 (17.8)	<0.001
White blood cells – ×10^9/L	5.6 (4.5-7.0)	5.7 (4.5-7.2)	5.3 (4.6-6.7)	5.1 (4.2-6.3)	0.127
Neutrophils – ×10^9/L	3.7 (2.7-5.2)	3.8 (2.8-5.4)	3.5 (2.9-4.9)	3.5 (2.6-4.6)	0.352
Lymphocyte – ×10^9/L	1.2 (0.9-1.5)	1.2 (1.0-1.6)	1.1 (0.9-1.5)	1.1 (0.8-1.3)	0.001
Total cholesterol – mg/dL	196.0 (167.0-222.0)	198.0 (172.0-226.0)	187.5 (160.8-213.8)	179.5 (152.8-212.8)	0.009
Albumin – g/L	36.7 (30.1-40.5)	38.9 (36.4-41.8)	30.3 (29.5-31.7)	25.6 (23.2-27.4)	<0.001
Brain MRI assessment – n (%)					
Bilateral necrosis	181 (45.5%)	132 (47.5%)	17 (44.7%)	32 (39.0%)	0.399
Involving ≥2 brain regions	73 (18.3%)	42 (15.1%)	10 (26.3%)	21 (25.6%)	0.040

	All Patients	Absent risk	Moderate risk	Severe risk	*P* values
Sample size – n	**N=398**	**N=278**	**N=38**	**N=82**	
Anti-RN treatment – n (%)					
Corticosteroids	176 (44.2%)	139 (50.0%)	12 (31.6%)	25 (30.5%)	0.002
Bevacizumab	84 (21.1%)	65 (23.4%)	4 (10.5%)	15 (18.3%)	0.149
None of the above	168 (42.2%)	102 (36.7%)	23 (60.5%)	43 (52.4%)	0.002
Time from RT to RN – yrs, median (IQR)	3.4 (2.5-6.2) (*n*=283)	3.4 (2.5-7.0) (*n*=207)	3.4 (2.7-6.0) (*n*=21)	3.4 (2.8-5.1) (*n*=55)	0.997
TNM stage – n (%)					0.880
I	5/272 (1.8%)	4/198 (2.0%)	0/22 (0.0%)	1/52 (1.9%)	
II	22/272 (8.1%)	16/198 (8.1%)	1/22 (4.5%)	5/52 (9.6%)	
III	138/272 (50.7%)	101/198 (51.0%)	14/22 (63.6%)	23/52 (44.2%)	
IV	107/272 (39.3%)	77/198 (38.9%)	7/22 (31.8%)	23/52 (44.2%)	
RT technique – IMRT, n (%)	156/261 (59.8%)	115/193 (59.6%)	10/20 (50.0%)	31/48 (64.6%)	0.533
Tumor RT dose – Gy, mean (SD)	69.1 (7.6) (*n*=266)	69.5 (5.6) (*n*=196)	66.9 (13.9) (*n*=20)	68.7 (10.5) (*n*=50)	0.331
Neck RT dose – Gy, mean (SD)	54.0 (22.8) (*n*=278)	55.4 (21.1) (*n*=203)	53.2 (23.6) (*n*=20)	48.9 (27.9) (*n*=55)	0.169
Chemotherapy – n (%)	231/277 (83.4%)	168/203 (82.8%)	14/20 (70.0%)	49/54 (90.7%)	0.107
Neoadjuvant	123/277 (44.4%)	95/203 (46.8%)	5/20 (25.0%)	23/54 (42.6%)	0.166
Concurrent	190/277 (68.6%)	138/203 (68.0%)	12/20 (60.0%)	40/54 (74.1%)	0.479
Adjuvant	18/277 (6.5%)	13/203 (6.4%)	2/20 (10.0%)	3/54 (5.6%)	0.764

Abbreviations: GNRI, Geriatric Nutritional Risk Index; PNI, Prognostic Nutritional Index; CONUT, Controlling Nutritional Status; BMI, Body Mass Index; SD, Standard deviation; IQR, Interquartile range; RT, Radiotherapy; RN, Radiation-induced brain necrosis; IMRT, Intensity-modulated radiation therapy; MRI, Magnetic resonance imaging.

Table S3. Baseline characteristics grouped by CONUT strata.

	All Patients	Absent risk	Mild risk	Moderate risk	Severe risk	P Values
Sample size – n	**398**	**99**	**187**	**90**	**22**	
Sex – males, n (%)	291 (73.1%)	76 (76.8%)	126 (67.4%)	70 (77.8%)	19 (86.4%)	0.080
Age – yrs, median (IQR)	50.9 (44.5-57.0)	50.2 (42.8-56.0)	50.7 (45.6-56.0)	51.7 (42.8-58.6)	54.9 (50.5-61.6)	0.106
Follow-up period– yrs, median (IQR)	2.3 (1.1-3.6)	2.1 (1.1-4.7)	2.3 (1.1-4.2)	2.5 (1.4-3.0)	2.4 (1.1-2.9)	0.430
Presence of primary endpoint – n (%)	42 (10.6%)	7 (7.1%)	16 (8.6%)	14 (15.6%)	5 (22.7%)	0.049
GNRI – point, median (IQR)	92.4 (83.1-99.5)	98.9 (96.2-102.9)	94.6 (88.0-100.2)	80.3 (75.5-84.2)	72.9 (70.7-75.7)	<0.001
GNRI strata						.
>98 – Absent risk	128 (32.2%)	60 (60.6%)	68 (36.4%)	0 (0.0%)	0 (0.0%)	
>92, ≤98 – Mild risk	77 (19.3%)	29 (29.3%)	47 (25.1%)	1 (1.1%)	0 (0.0%)	
>82, ≤92 – Moderate risk	104 (26.1%)	10 (10.1%)	60 (32.1%)	34 (37.8%)	0 (0.0%)	
≤82 – Severe risk	89 (22.4%)	0 (0.0%)	12 (6.4%)	55 (61.1%)	22 (100.0%)	
PNI – point, median (IQR)	42.6 (36.5-47.2)	48.1 (46.0-51.9)	43.0 (40.6-46.6)	33.7 (31.3-36.3)	27.8 (26.2-29.3)	<0.001
PNI strata						.
>38 – Absent risk	278 (69.8%)	99 (100.0%)	167 (89.3%)	12 (13.3%)	0 (0.0%)	
>35, ≤38 – Moderate risk	38 (9.5%)	0 (0.0%)	19 (10.2%)	19 (21.1%)	0 (0.0%)	
≤35 – Severe risk	82 (20.6%)	0 (0.0%)	1 (0.5%)	59 (65.6%)	22 (100.0%)	
CONUT – point, median (IQR)	3.0 (2.0-5.0)	1.0 (0.0-1.0)	3.0 (2.0-3.0)	6.0 (5.0-7.0)	9.0 (9.0-10.0)	<0.001
Height – cm, mean (SD)	165.4 (7.3)	165.6 (7.2)	164.8 (7.3)	165.9 (7.5)	167.5 (5.7)	0.318
Weight – kg, mean (SD)	59.1 (10.7)	61.4 (11.0)	58.0 (10.7)	59.0 (10.8)	58.6 (7.6)	0.091
BMI – kg/m^2, mean (SD)	21.5 (3.2)	22.3 (3.2)	21.2 (3.0)	21.4 (3.5)	20.9 (2.7)	0.045
Tumor progression before RN – Yes, n (%)	47 (11.8%)	7 (7.1%)	24 (12.8%)	14 (15.6%)	2 (9.1%)	0.295
Lower cranial nerves injury – Yes, n (%)	177 (44.5%)	39 (39.4%)	85 (45.5%)	38 (42.2%)	15 (68.2%)	0.098
Hypertension – Yes, n (%)	47 (11.8%)	13 (13.1%)	22 (11.8%)	8 (8.9%)	4 (18.2%)	0.579
Diabetes – Yes, n (%)	15 (3.8%)	4 (4.0%)	9 (4.8%)	2 (2.2%)	0 (0.0%)	0.734
Stroke – Yes, n (%)	33 (8.3%)	6 (6.1%)	17 (9.1%)	8 (8.9%)	2 (9.1%)	0.800
Cigarette Smoking – Yes, n (%)	57 (14.3%)	13 (13.1%)	29 (15.5%)	12 (13.3%)	3 (13.6%)	0.965
Alcohol consumption – Yes, n (%)	24 (6.0%)	4 (4.0%)	12 (6.4%)	6 (6.7%)	2 (9.1%)	0.686
Laboratory tests – median (IQR)						
Red blood cells – ×10^9/L	4.4 (4.1-4.8)	4.5 (4.2-4.9)	4.4 (4.2-4.8)	4.3 (3.9-4.7)	4.1 (3.7-4.5)	0.001
Hemoglobin – g/L	129.1 (16.5)	133.0 (14.2)	130.5 (15.8)	124.4 (16.2)	118.5 (23.8)	<0.001
White blood cells – ×10^9/L	5.6 (4.5-7.0)	6.5 (5.4-8.4)	5.3 (4.3-6.7)	5.3 (4.3-6.6)	5.3 (4.1-7.7)	<0.001
Neutrophils – ×10^9/L	3.7 (2.7-5.2)	4.0 (2.9-5.6)	3.6 (2.7-4.8)	3.5 (2.7-4.8)	3.8 (2.4-5.3)	0.155
Lymphocyte – ×10^9/L	1.2 (0.9-1.5)	1.7 (1.4-2.0)	1.0 (0.9-1.3)	1.1 (0.9-1.4)	0.9 (0.6-1.1)	<0.001
Total cholesterol – mg/dL	196.0 (167.0-222.0)	213.0 (186.0-232.5)	197.0 (168.0-222.0)	180.0 (158.0-212.0)	139.5 (132.8-167.2)	<0.001
Albumin – g/L	36.7 (30.1-40.5)	39.9 (37.8-42.7)	37.9 (33.9-41.2)	28.0 (25.7-30.1)	23.4 (21.1-24.5)	<0.001
Brain MRI assessment – n (%)						
Bilateral necrosis	181 (45.5%)	55 (55.6%)	83 (44.4%)	34 (37.8%)	9 (40.9%)	0.090
Involving ≥2 brain regions	73 (18.3%)	14 (14.1%)	34 (18.2%)	20 (22.2%)	5 (22.7%)	0.472
Anti-RN treatment – n (%)						

	All Patients	Absent risk	Mild risk	Moderate risk	Severe risk	P Values
Sample size – n	**398**	**99**	**187**	**90**	**22**	
Corticosteroids	176 (44.2%)	55 (55.6%)	85 (45.5%)	29 (32.2%)	7 (31.8%)	0.008
Bevacizumab	84 (21.1%)	22 (22.2%)	45 (24.1%)	12 (13.3%)	5 (22.7%)	0.201
None of the above	168 (42.2%)	31 (31.3%)	77 (41.2%)	49 (54.4%)	11 (50.0%)	0.012
Time from RT to RN – yrs, median (IQR)	3.4 (2.5-6.2) (*n*=283)	3.9 (2.5-8.2) (*n*=75)	3.3 (2.3-5.6) (*n*=133)	3.5 (3.0-5.5) (*n*=59)	3.0 (2.1-4.6) (*n*=16)	0.197
TNM stage – n (%)						.
I	5/272 (1.8%)	2/72 (2.8%)	2/129 (1.6%)	1/55 (1.8%)	0/16 (0.0%)	
II	22/272 (8.1%)	5/72 (6.9%)	11/129 (8.5%)	5/55 (9.1%)	1/16 (6.2%)	
III	138/272 (50.7%)	38/72 (52.8%)	66/129 (51.2%)	25/55 (45.5%)	9/16 (56.2%)	
IV	107/272 (39.3%)	27/72 (37.5%)	50/129 (38.8%)	24/55 (43.6%)	6/16 (37.5%)	
RT technique – IMRT, n (%)	156/261 (59.8%)	37/67 (55.2%)	77/126 (61.1%)	34/54 (63.0%)	8/14 (57.1%)	0.816
Tumor RT dose – Gy, mean (SD)	69.1 (7.6) (*n*=266)	69.4 (4.6) (*n*=70)	69.5 (6.0) (*n*=127)	67.7 (13.0) (*n*=55)	70.1 (1.5) (*n*=14)	0.473
Neck RT dose – Gy, mean (SD)	54.0 (22.8) (*n*=278)	51.5 (23.5) (*n*=74)	58.1 (18.5) (*n*=129)	49.4 (27.0) (*n*=59)	49.2 (29.6) (*n*=16)	0.044
Chemotherapy – n (%)	231/277 (83.4%)	56/74 (75.7%)	110/129 (85.3%)	51/58 (87.9%)	14/16 (87.5%)	0.243
Neoadjuvant	123/277 (44.4%)	36/74 (48.6%)	57/129 (44.2%)	22/58 (37.9%)	8/16 (50.0%)	0.630
Concurrent	190/277 (68.6%)	45/74 (60.8%)	91/129 (70.5%)	44/58 (75.9%)	10/16 (62.5%)	0.261
Adjuvant	18/277 (6.5%)	5/74 (6.8%)	9/129 (7.0%)	3/58 (5.2%)	1/16 (6.2%)	0.980

Abbreviations: GNRI, Geriatric Nutritional Risk Index; PNI, Prognostic Nutritional Index; CONUT, Controlling Nutritional Status; BMI, Body Mass Index; SD, Standard deviation; IQR, Interquartile range; RT, Radiotherapy; RN, Radiation-induced brain necrosis; IMRT, Intensity-modulated radiation therapy; MRI, Magnetic resonance imaging.

Figure S1. Baseline GNRI and post-RN survival.

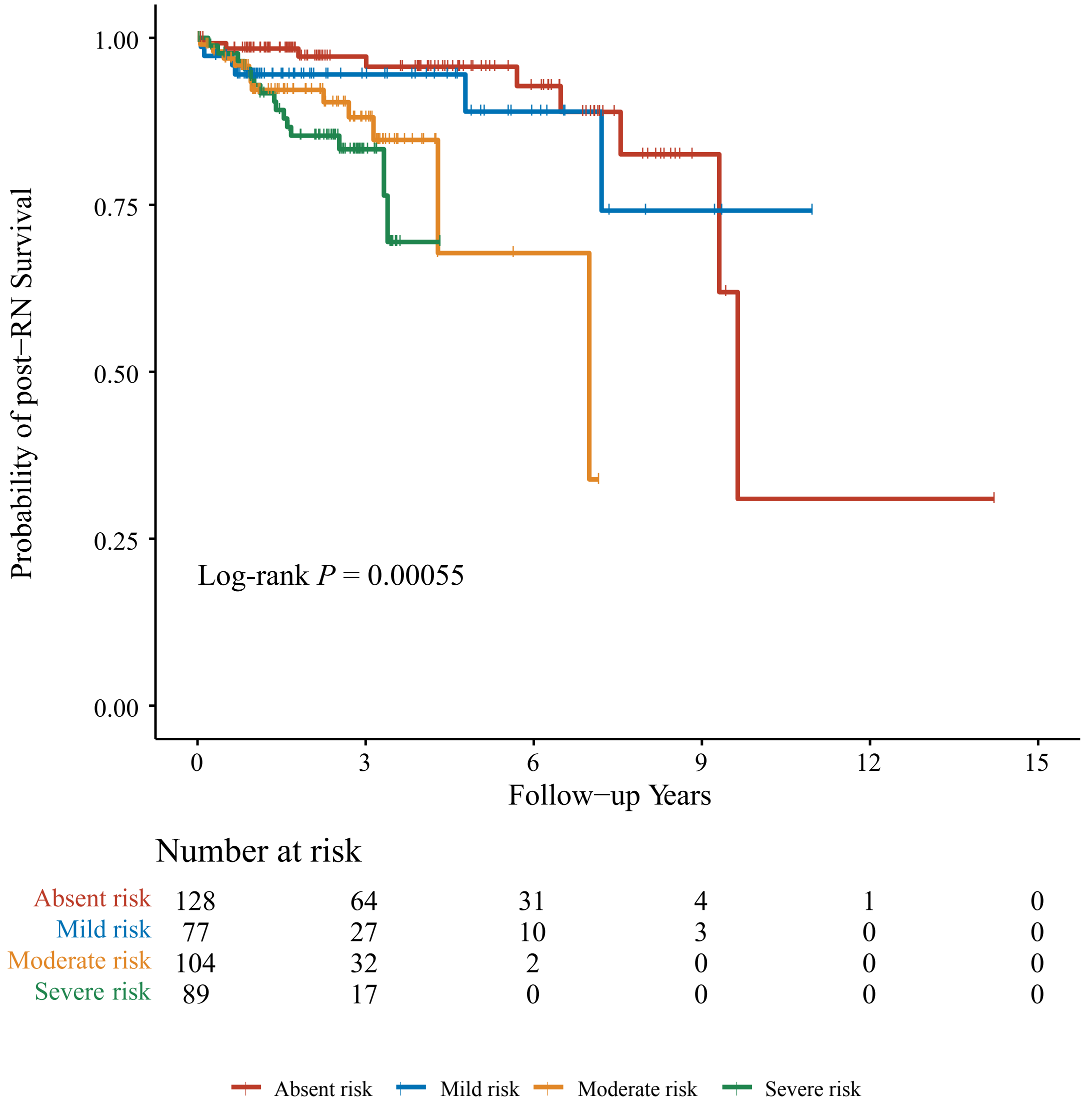

Figure S2. Baseline PNI and post-RN survival.

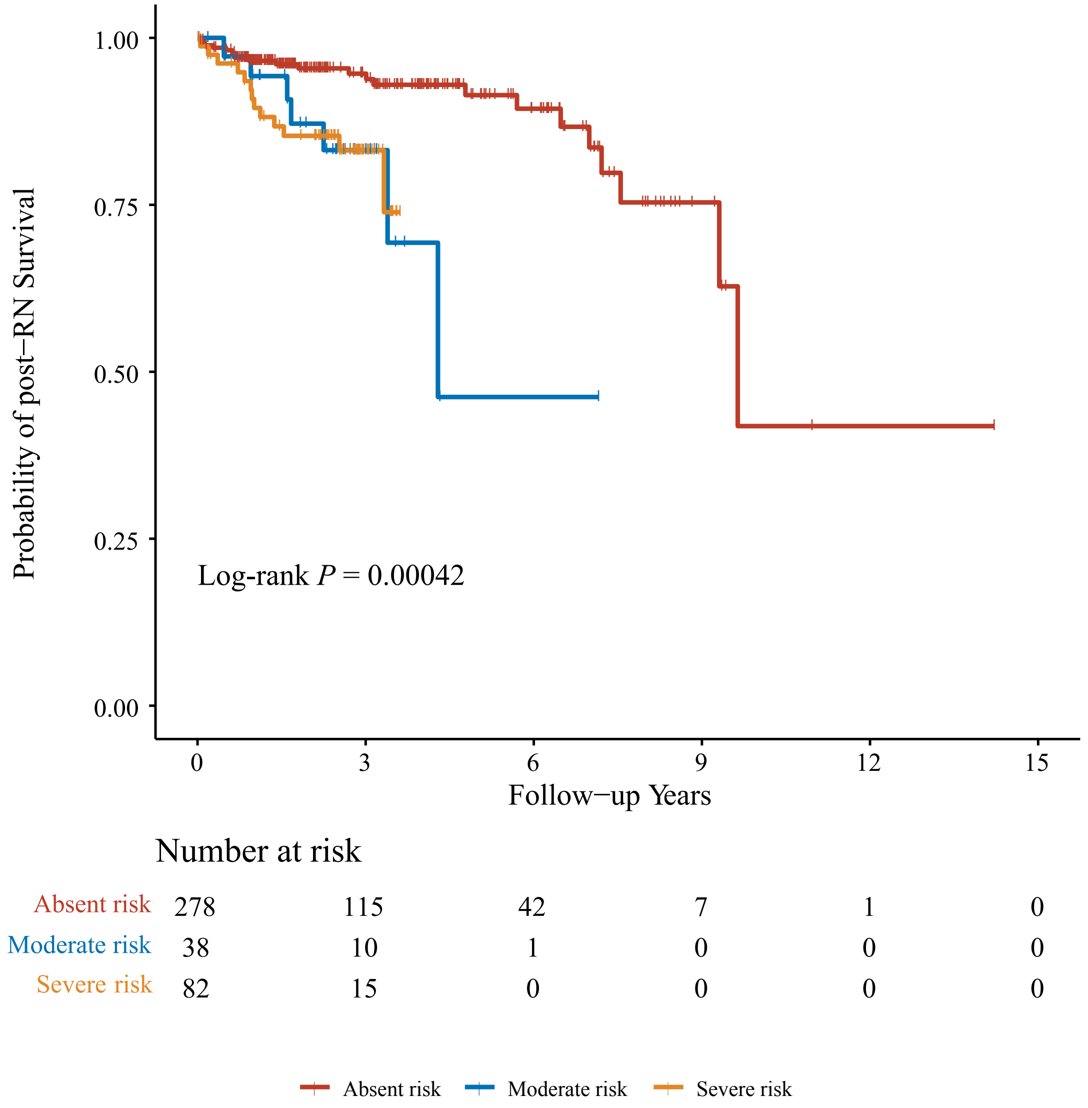

	0	3	6	9	12	15
Absent risk	278	115	42	7	1	0
Moderate risk	38	10	1	0	0	0
Severe risk	82	15	0	0	0	0

Figure S3. Baseline CONUT and post-RN survival.

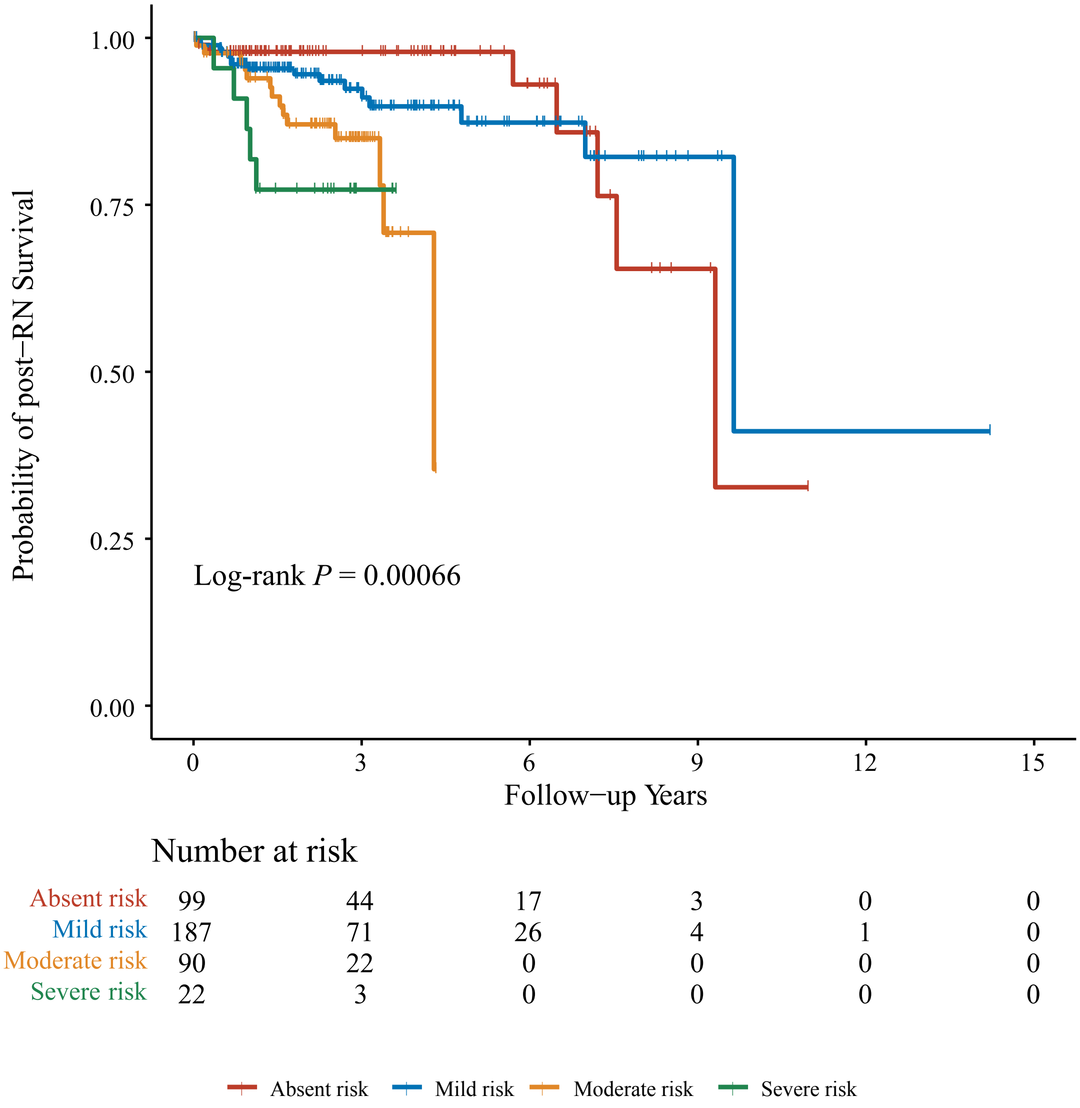

Figure S4. The optimal cut-off value of GNRI determined by the X-tile software.

The X-tile identified a cut-off point of 92.7 for GNRI to categorize patients into either high- or low-risk groups (maximum χ^2=16.9502, P=00016).

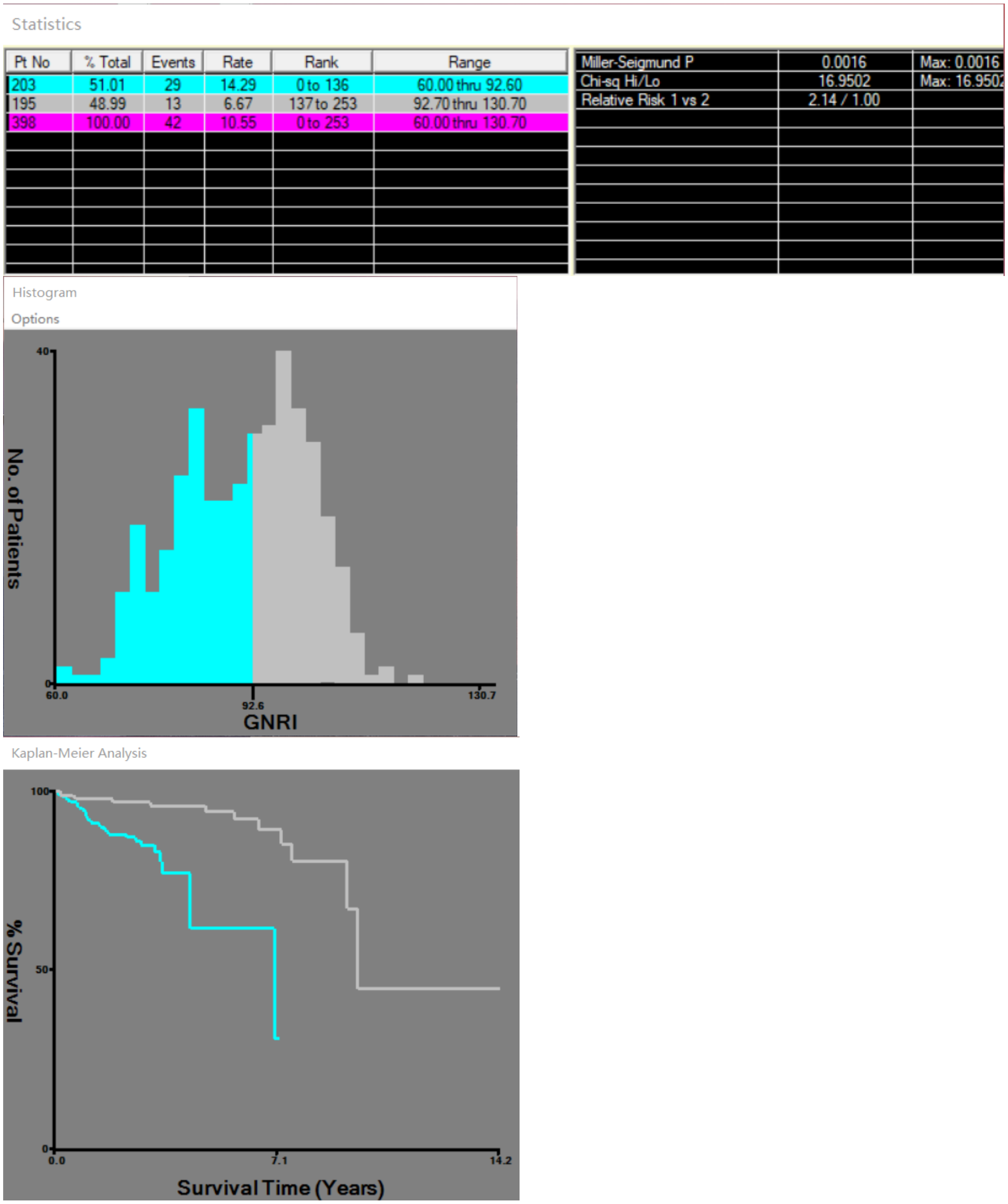

Figure S5. The optimal cut-off value of PNI determined by the X-tile software.

The X-tile identified a cut-off point of 43.7 for PNI to categorize patients into either high- or low-risk groups (maximum χ^2=20.9658, P<0.0001).

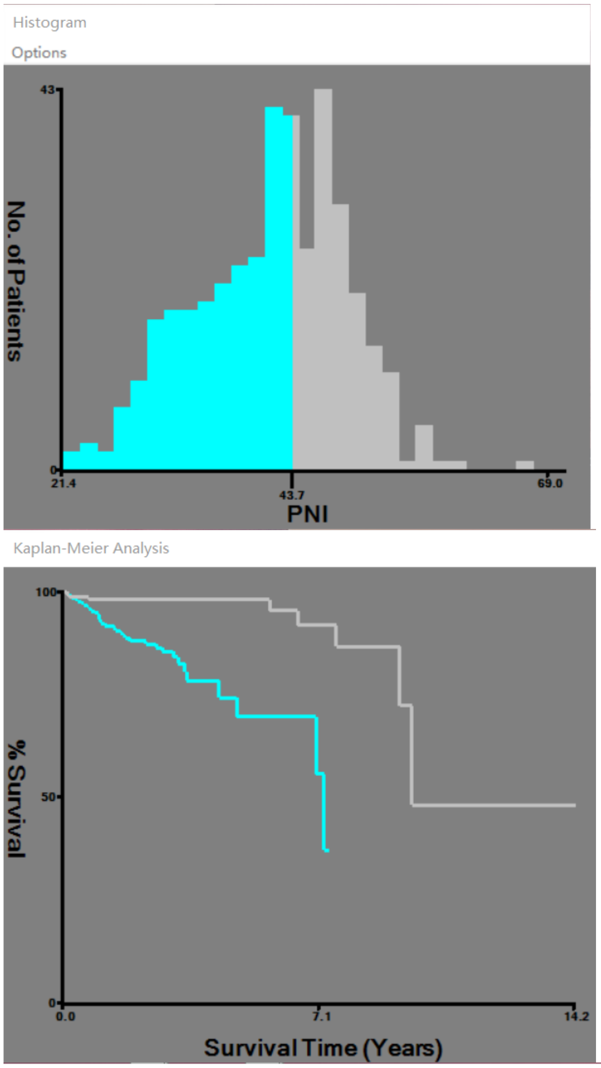

Statistics

Pt No	% Total	Events	Rate	Rank	Range
230	57.79	34	14.78	0 to 167	21.35 thru 43.65
168	42.21	8	4.76	168 to 284	43.80 thru 69.00
398	100.00	42	10.55	0 to 284	21.35 thru 69.00

Miller-Seigmund P	<0.0001	Max: <0.000
Chi-sq Hi/Lo	20.9658	Max: 20.965
Relative Risk 1 vs 2	3.10 / 1.00	

Figure S6. The optimal cut-off value of CONUT determined by the X-tile software.

The X-tile identified a cut-off point of 92.7 for CONUT to categorize patients into either high- or low-risk groups (maximum χ^2=14.3349, P=0.0053).

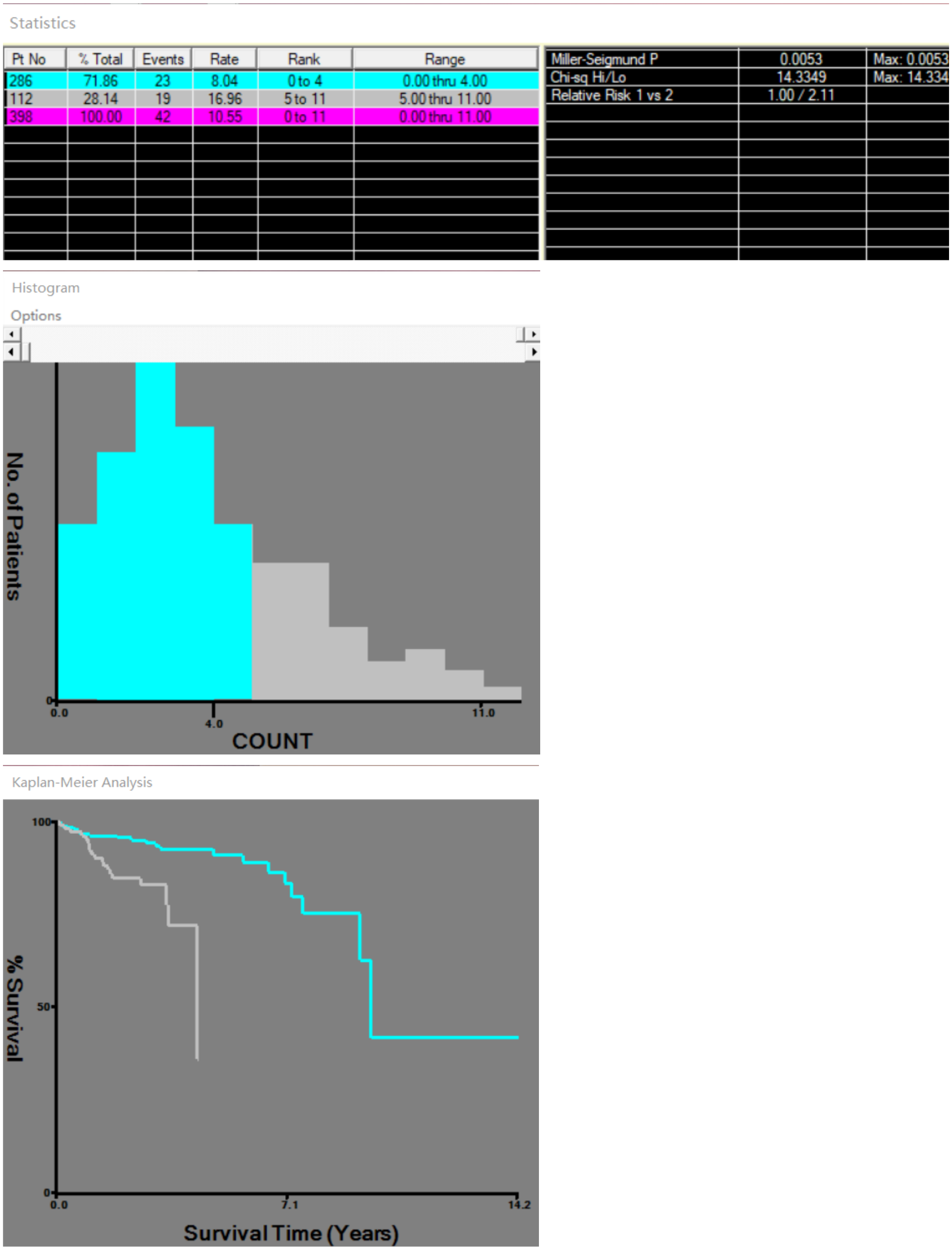

Supplementary analysis. The prognostic value of the baseline BMI in predicting mortality in head and neck cancer survivors with radiation-induced brain necrosis.

**BMI**		Model 0		Model 1		Model 2	
		Unadjusted-HR (95% CI)	*p* values	Adjusted-HR (95% CI)	*p* values	Adjusted-HR (95% CI)	*p* values
Normal	(18.5≤BMI<24)	[Ref]	··	[Ref]	··	[Ref]	··
Slim	(BMI<18.5)	2.25 (1.10-4.59)	0.03	2.27 (1.07-4.81)	0.03	3.43 (1.34-10.34)	0.03
Overweight	(24≤BMI<28)	1.34 (0.61-2.92)	0.47	1.23 (0.54-2.79)	0.62	0.71 (0.21-2.44)	0.58
Obese	(BMI⩾28)	incalculable	>0.99	incalculable	>0.99	incalculable	>0.99
BMI per 1-point increased		0.88 (0.79-0.98)	0.02	0.89 (0.80-0.997)	0.04	0.84 (0.72-0.98)	0.03

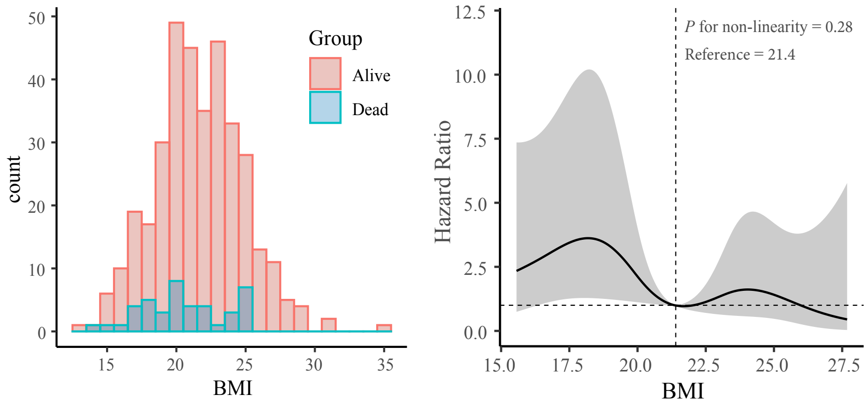

Supplement: Supplementary file 1 [file nutrients-15-01973-s001.zip › nutrients-2235929-supplementary.pdf]
